# Cina

**Published:** 1892-04

**Authors:** Thos. Wildes

**Affiliations:** Kingston, Jamaica


### CINA.

THOS. WILDES, M. D., KINGSTON, JAMAICA.

I desire to call attention to Cina for some cases of chronic parenchymatous nephritis, with some or all the symptoms, as follow:

Gout; rheumatism; urine reddish-brown, loaded with uric acid, and stinking outrageously, like rotten eggs, or rotten meat, or chicken-dung, or all; patient irritable, nervous, erratic, and highly excited by ardent spirits; cerebral hyperæmia; miserable, restless sleep; even unable to lie in bed, but must sit in chair or walk; wakefulness for hours, with agonizing apprehensions and palpitation of heart, with oppression, fullness, pressure, and pain in region of heart; great depression of spirits.

It is seldom that Cina would be the first remedy thought of for such symptoms, but after all other remedies have failed, it will create a complete metamorphosis within thirty-six hours, and cause the disappearance of a multiplicity of disagreeable symptoms.

Even for the pathological condition of the kidney, Cina will be found to be the main remedy.

The guiding symptom for Cina is the INFAMOUS ODOR OF THE URINE, EVEN WHEN FIRST PASSED, AND INCREASED BY STANDING.

In all conditions, as above described, there co-exists hyperæmia of the base of the brain, either active or passive.

